# Office Visitors

**Published:** 1907-08

**Authors:** 


### OFFICE VISITORS.

He stalked into our summer office--a long tall gaunt saturnine mountaineer with long hair and long moustache. The very picture seen in Northern illustrated print, as the typical Southerner. "Why hello Doc, when did yer get up from down south?" (Every one is from down south that comes to the Blue Ridge Mountains, no matter where his home is.) "I'm mity glad ter see yer, I run in jest es soon es I knowed yer wus yere, to see yer and shake wid yere, an ter tell yer that them teeth yer put in fer me las summer is ther bes things I ever paid money fer, sense I've abin takin kere of mysef. Yes sur, I haint bit ento, ner chawed er apple, now gwine on ten yere, till I got them teeth, an now I'm all rite, sure an yer know apples is fruit wid us mountain fokes. I'd like fer yer jest ter look at em er bit, I cum down on er grit een mer terbacker tother day an afterwards I felt em sorter ruff on ther aige, I reckun I sorter frazed em sum. All rite, jest smooth em off er leettle—I

thot ther aige of them back teeth was er leetle slivered. Say Doc, have yer got any of thet red liquor like yer hed las summer wen yer pulled mer ole snags. I wants yer ter jerk thet ole wisdum tooth back thar I would'nt let yer pull wen yer wus erpullin them tothers. He is on ther lower jaw an loose now but I kaint stan it widdout er drink." (South of Mason & Dixon's line "a drink" means one of whiskey—any other sort is qualified with the name of what is desired—a drink of water, or beer, or cider.) "Ah! thets good stuff, bettern we kin git about yere now-er-days. No I don't want none er thet cocaine pizen stuff dejected eento my jaw. Er tooth-dentis kilt er woman down een Souf Carlina, hit haint bin moren er month er two ergo, an I don't want nun sich een me. Jest yer clap on them pullikins, take er good holt now, Doc, an git hit outer thar. Ouch! Goramity! yer got hit sho pop. I'd er hollered ef hit hadn't er bin fur ther dram. Thet was slick dun, Doc, Gimme er leetle mo er the red stuff ter take ther tase of ther blood out er mer mouf, jest er swaller. We kaint git good licker now, Doc, no, down een ther dark corner of yon county we could git hit mos eny time, an good stuff till erbout er yere ergo. The fokes stood by ther stillers, an ther revenoo men wus helt off and misled and ther wus nun tuck; but here er late ther fellers got too honkerish after money fur hit, an got to dulteratin hit wid consecrated lye an sich, an hit got ther best of them thet drunk hit, an hit made ther boys crazy like, an ther wus too many fites, an sum killin. So ther fokes got tired of that ar an give em away ter ther revenoo, and tha cum an everlastin busted ther stills up, spilt ther mash an stuff, an tuck em off ter jail. So we'uns kaint git nothin thets good no mo lessin we sen off ter Asheville fur hit. My cousin Sally Ann Shouter, ther prechers wife, is er talkin of comin ter see yer about her mouf. She lows that if you'll make her a set es good es mine she would'nt mind spendin ther money. I told her yer never made but one rele puffeck set natully, an that was mine, but she's er comin. Well I must go. Cum down an take er fish een ther river for ther trout. Ther squirrels will soon be ripe too, they is begin ter cut on ther hickory nuts er little alreddy." We urged another swallow of red rye and shook his honest hard palm.

